# Evaluation of spray and oral delivery of Newcastle disease I2 vaccine in chicken reared by smallholder farmers in central Ethiopia

**DOI:** 10.1186/s12917-018-1355-x

**Published:** 2018-02-13

**Authors:** Kibrom Mebrahtu, S. Teshale, Wendimeneh Esatu, Tadios Habte, Esayas Gelaye

**Affiliations:** 1 Jinka Agricultural Research Center, Department of Animal Health, Southern Ethiopian Nations, Nationalities and People’s Regional State, Jinka, Ethiopia; 2 Addis Ababa University College of Veterinary Medicine and Agriculture, Bishoftu Ethiopia, Department of Clinical Studies, P.O. Box 34 Bishoftu, Ethiopia; 3 Ethiopian Institute of Agriculture, Debre Zeit Agricultural Research Center, Bishoftu, Ethiopia; 4 National Veterinary Institute, Bishoftu, Ethiopia

**Keywords:** Newcastle disease, I2 vaccine, Smallholder, Village, Chicken

## Abstract

**Background:**

Newcastle disease (ND) is a highly infectious disease causing considerable economic losses to poultry farmers worldwide. Conventional vaccine delivery methods are not suitable for smallholder and rural poultry producers, and thus appropriate vaccination methods need to be sought. This study was carried out with the main objective of evaluating the efficacy of ND I2 vaccine delivered via drinking water and spray under smallholder farmers’ condition in Minjar-Shenkora district, central Ethiopia. Twenty households were randomly assigned to intervention and control groups. Chickens owned by the selected households were randomly assigned to one of the three intervention groups. Blood samples were collected regularly for antibody assay from individual chicken vaccinated with ND I2 vaccine using different routes.

**Results:**

At baseline, there was no difference in antibody titer among the experimental groups. After the first and booster vaccinations, the three vaccinated groups had significantly higher antibody titer (*P* < 0.001) than the unvaccinated control group. Interestingly, there was no statistically significant difference in antibody titer among the vaccinated groups. Out of the 40 chicken in the unvaccinated control only 14 had antibody titter≥ log_2_^3^. Similarly 19/37 of chicken in the drinking water group, 19/37 of chicken in the eye drop group and 20/40 chicken in the spray group had antibody titer ≥ log_2_^3^. Two weeks after the first vaccination the proportion of chicken with antibody titer ≥ log_2_^3^ rose to 23/37, 30/37 and 29/40 in the group vaccinated via drinking water, eye drop and spray, respectively. The proportion remained low in unvaccinated group. Hundred percent of the vaccinated chicken survived after infection with the virulent ND virus (Alemaya strain); whereas only 40% survived from the unvaccinated control group.

**Conclusion:**

The results of this study showed that ND I2 vaccine administered via drinking water and spray under smallholder farmers’ situation provoked protective antibody level similar to the eye drop method. The use of ND I2 vaccine could contribute to food security if used by rural poultry farmers properly.

## Background

Chicken production is considered an important source of quality animal proteins and can break the vicious cycle of poverty and malnutrition in developing countries. They particularly play important role in the well-being of women and children [1]. In Ethiopia, locally produced chickens are genetically diverse [2] with low production [3], which may be attributed to ND.

Increasing productivity and financial return from backyard chicken requires better knowledge of diseases such as ND. Newcastle disease can intercept the expansion of small scale poultry farms in the rural Ethiopia unless its incidence is reduced. Distribution of various breeds and uncontrolled marketing can aid the spread of ND between and within poultry farms. For example, more than 15 outbreaks of ND were confirmed in 2016 whereas as hundreds of outbreaks were confirmed in 2015 in the country. This shows that ND is continuing to impact the livelihood of poultry farmers. To improve productivity and financial return from backyard flocks, improvements to education and biosecurity measures are required.

Effective control of ND is needed to improve the livelihood of smallholder farmers particularly women in developing countries [4]. Vaccination has been considered effective and affordable control option in several countries [4–8]. It has been widely used in commercial poultry farms. The demand for vaccine against ND has grown in smallholder and village poultry producers. However, the conventional vaccine application methods are not suitable for smallholder and village poultry production systems [6]. An innovative alternative for smallholder and village poultry producers was developed in Australia using heat stable ND I2 virus [7]. It has been proven to be suitable for village poultry in Asian and some African countries in the absence of cold chain [9] and is cheaper than conventional ND vaccines [8]. Despite expansion of smallholder poultry production, information on the use of ND I2 vaccine and route of vaccine delivery with optimal protection level has not been sufficiently explored in Ethiopia. Suitable vaccine delivery route that can be used by the farmers could contribute to food security. The main objective of this study is, therefore, to evaluate the efficacy of ND I2 vaccine delivered via drinking water and spray.

## Methods

### Study area

**Fig. 1:**
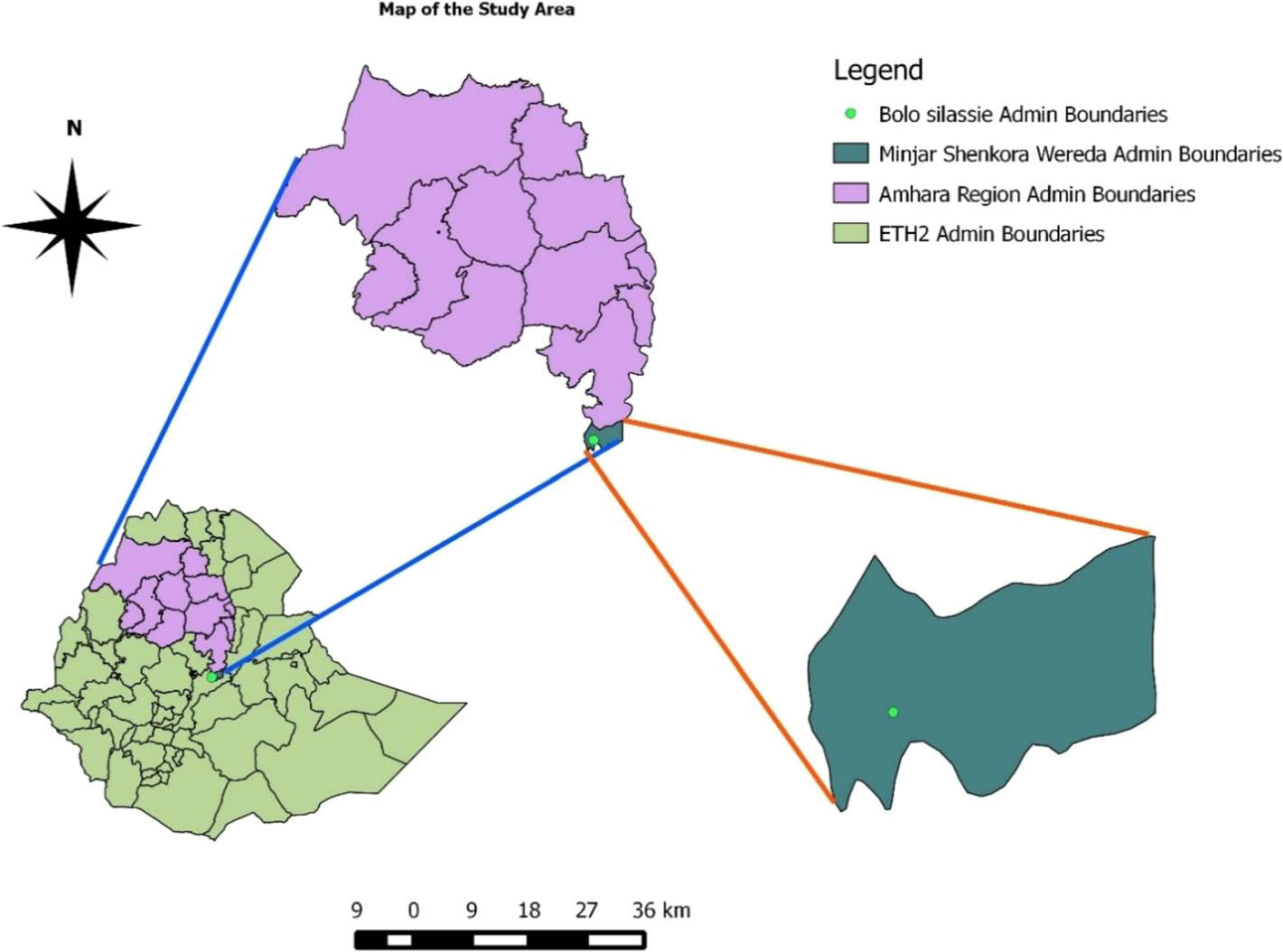
Map of the study area (Quantum Geographic Information System (QGIS) software Version 2.0.1)

This study was conducted in Minjar-Shenkora district, North Shoa Zone, Amhara Regional State, central Ethiopia. It is located between 90^°^6′ and 90^°^5’ N and 39^°^46′ and 39^°^26′ East 135 km south east of Addis Ababa (Fig. 1). The altitude of the area ranges from 1400 to 2400 m.a.s.l. The average annual rainfall of the district ranges from 62.8 mm – 1028 mm in bimodal pattern. The mean annual temperature of the last 10 years ranges from 7.3 °C – 20 °C. Agriculture is the main stay of the economy of people inhabiting the district in which livestock production plays key role. The livestock population of the district comprises cattle (95270), sheep (57603), goats (74049), poultry (168,702) and equines (3826). Exotic poultry such as Sasso, Kockock and White leghorn are widely reared in the district although the majority is made of indigenous chicken (Chefe ecotype) [10]. Chickens in the study area have never been vaccinated against ND.

### Study methods

Minjar-Shenkora district was purposively selected for this study. It has several peasant associations, which are the smallest administrative units within the district. The peasant associations were selected purposively considering the presence of higher chicken population per household, the accessibility of the peasant association to the road and the consent of the farmers to participate in the study. The selection of peasant association was facilitated by livestock experts and extension staff of the district. Households from the selected peasant associations were selected purposively based on the number of indigenous and exotic chickens they own. This was facilitated by community leaders and extension staffs. Accordingly 20 households who owned ten or more chicken (indigenous and exotic) per household were selected and included in the experiment. A total of 154 4 weeks old chickens, 82 indigenous and 72 exotic breeds owned by the 20 selected households were selected and individually identified using numbered wing tags. The farmers are smallholder farmers who use open housing and small areas enclosed with fence. The feed resources for the chickens are household refuse, homestead pickings, crop residues and seeds offered by the flock owners. This study was submitted to the Animal Research Ethics Review Committee and approved.

### Experimental design

The selected households were randomly assigned to either intervention or control groups. The chicken owned by the households assigned to intervention were randomly assigned to one of the three intervention groups: the first group comprises of chicken which received ND I2 vaccine via drinking water, the second group comprises of chicken which received ND I2 vaccine via spray and the last group comprises of chicken which received ND I2 vaccines via eye drop (positive control). The chickens were stratified by their breeds. Randomization of households was carried out using lottery systems whereas that of chickens was carried out by random sampling method using R statistical software. The unvaccinated control group and the group that received ND I2 via spray comprise 40 chickens each (22 indigenous and 18 exotic chicken per group) whereas the other two groups had 37 chickens each (19 indigenous and 18 exotic chickens per group). Blood sample was collected to estimate the baseline antibody level against ND virus.

### Vaccination and follow up

Vials of 400 and 200 doses of freeze dried ND I2 vaccine (batch number NVI-4/16 and 1/17, respectively) containing 10^6^ EID_50_ viruses were purchased from the National Veterinary Institute (NVI), Bishoftu, Ethiopia. The vaccine was reconstituted with non-chlorinated distilled water (manufacturer’s instruction) and given to experimental chickens except the unvaccinated group. Booster vaccination was given 15 days after the first vaccination using the same vaccine. Blood samples were collected 15 days after the first and booster vaccinations. The chickens were followed for 3 months after which they were infected with virulent ND virus. The vaccine delivery was carried out by veterinary technicians in the district who were not aware of the experiment.

For oral delivery via drinking water a vial of the freeze-dried ND I2 vaccine containing 400 doses was reconstituted in 4000 mL of distilled water following the manufacturer’s instructions (1 dose per 10 mL of distilled water). Each chicken per group received 10 mL of the reconstituted vaccine. Prior to vaccination farmers were informed to withhold water for few hours. The chickens were provided with reconstituted vaccine individually. For ocular administration a vial of the freeze-dried ND I2 vaccine containing 200 doses was reconstituted in 10 mL of saline solution as recommended by the manufacturer. Individual chicken in the group was provided with one drop of the reconstituted vaccine using sterile pipette. For the group receiving ND I2 vaccine by spray a spray bottle was used to spray the vaccine in saline water. In this group hundred mL of saline water was used to reconstitute 100 dose of the vaccine as per the recommendation of the manufacturer. Therefore, one vial of the freeze-dried ND I2 vaccine containing 400 doses was reconstituted in 400 mL of saline water. That is, the dose of the vaccine was adjusted at 1 mL of vaccine per chicken in a cage and the chickens were kept in closed cage for 30 min after spray.

### Blood collection

Blood samples (1–1.5 mL) per bird were collected using 3 mL sterile disposable syringe from the wing vein following the standard methods described by Alders and Spradbrow [6]. The collected blood samples were labeled with individual chicken number and allowed to clot overnight at room temperature to facilitate serum separation. The sera were harvested in to labeled cryovials and stored at − 20 °C until HI was carried out. Collection of the blood for serological testing was carried out by the district veterinary technicians.

### Hemagglutination inhibition (HI) assay

HI assay was conducted in the serology laboratory of the NVI, Bishoftu, Ethiopia by personnel who were not aware of the groups of chickens. The sera samples were heat inactivated at 56 °C for 30 min before assay. The level of anti-ND virus antibodies in the sera was estimated using the HI test as described by OIE [11]. The antigen used was reconstituted commercial NDV La Sota vaccine (TAD, Cuxhaven, Germany). Two-fold serial dilutions of serum samples were used to estimate the anti-NDV antibody titers as logarithms to the base two. In this study, we used the published cut off value for the protective HI antibody titer (HI titer ≥ log_**2**_^**3**^ i.e. GM ≥ 3) for ND vaccination in chickens [9, 11, 12].

### Challenge infection with virulent virus

Four weeks after booster vaccination was provided 40 chickens (10 from each treatment group) were randomly selected, purchased and brought to the College of Veterinary Medicine Addis Ababa University, Bishoftu, Ethiopia. The chickens were housed in experimental poultry house. They were infected with local virulent NDV strain designated Alemaya strain obtained from the NVI, Bishoftu, Ethiopia. It has a mean embryonic death time of 51.1 h, an intracerebral pathogenicity index of 1.84 and an intravenous pathogenicity index of 2.51 [13]. Each bird was inoculated with 1 mL of the viral suspension containing 10^7^ EID_50/_mL via breast muscle as described by Reta et al. [14]. The chickens from each treatment group were kept separately and followed daily for morbidity and mortality for 1 month. The birds were kept in experimental poultry house that is isolated from poultry and other livestock farms under the auspice of the National Veterinary Institute, Bishoftu, Ethiopia. The experimental house is equipped with necessary containment facility.

### Data analysis

Sample size calculation was done taking into account 5% error, 80% power and the expected difference among the group to be 0.75 units. Accordingly at least 28 chickens were needed per group. The data collected during this study were analyzed using STATA version 13. The variation in mean antibody titres among the four experimental groups was analyzed using one way ANOVA at each sampling time. When difference was observed among the groups, Bonferroni multiple pair-wise comparison was used and significance is reported at *P* < 0.05. For all analysis intention-to-treat analysis was used.

## Results

### Results of Hemaglutination inhibition assay

At baseline the overall mean (log_**2**_ ± SE) antibody titer against NDV was 3.9 ± 0.21, which was slightly higher than the protective level in all the treatment groups (Table 1). At this time there was no statistically significant difference in antibody titer among chicken in all the experimental groups. There was no statistically significant difference in mean antibody titers between indigenous and exotic chickens. However, the mean antibody titer was higher in exotic breeds than the indigenous chickens.

**Table 1:** The baseline and post-vaccination mean ± SE antibody titer of chicken in all experimental groups

Treatment Group	M ± SE HI antibody titer (log2) of chickens vaccinated by different methods			
	N	Day 0	Day 15	Day 31
Unvaccinated	40	3 ± 0.41	4.45 ± 0.58	4.4 ± 0.60
Drinking water	37	4.7 ± 0.39	7 ± 0.51	7.6 ± 0.38
Eye drop	37	4.1 ± 0.38	7.2 ± 0.41	7.1 ± 0.41
Spray	40	3.7 ± 0.46	8.05 ± 0.43	7.7 ± 0.41

Two weeks after the first vaccination the three vaccinated groups had significantly higher (*P* < 0.001) mean (log_**2**_ ± SE) antibody titer than the unvaccinated ones (Table 2). However, there was no statistically significant difference in antibody titer among the vaccinated groups although the group that received the ND I2 vaccine via spray had highest titer followed by the eye drop group. Similarly, after booster vaccination was provided the vaccinated groups had significantly higher antibody titer than the unvaccinated groups whereas there was no statistically significant difference among the vaccinated groups (Table 3). Highest antibody titer was observed in chickens vaccinated by spray followed by those that received the vaccine via drinking water.

**Table 2:** Results of multiple pair-wise comparison of log_2_ HI antibody titer in experimental chicken at day 15 after primary vaccination

Treatment Group-1	Treatment Group-2	MD	SE	*P*- value	95% CI
D. water^b^	Control^a^	2.55	0.705	0.002	0.66–4.43
Eye drop^b^	Control^a^	2.82	0.705	0.001	0.93–4.70
Spray^b^	Control^a^	3.6	0.691	0.000	1.75–5.44
Eye drop^b^	D. water^b^	0.27	0.718	1.000	−1.65 - 2.19
Spray^b^	D. water^b^	1.05	0.705	0.831	−0.83 - 2.93
Spray^b^	Eye drop^b^	0.77	0.705	1.000	−1.10 - 2.66

Groups with different letters significantly differ from each other; MD = mean difference; SE **=** standard error; D. water = drinking water

**Table 3:** Results of multiple pair-wise comparison of log_2_ HI antibody titer in experimental chicken at day 15 after booster vaccination

Treatment Group-1	Treatment Group-2	MD	SE	*P*-value	95% CI
D. water^b^	Control^c^	3.22	0.662	0.000	1.45–4.99
Eye drop^b^	Control^c^	2.73	0.662	0.000	0.96–4.50
Spray^b^	Control^c^	3.32	0.649	0.000	1.58–5.06
Eye drop^b^	D. water^d^	−0.48	0.675	1.000	−2.29 - 1.31
Spray^b^	D. water^d^	0.10	0.662	1.000	−1.66 - 1.87
Spray^b^	Eye drop^d^	0.66	0.662	1.000	−1.18 - 2.36

Groups with different letter significantly differ from each other; MD: mean difference; D. water = drinking water

### Proportion of chickens with protective (Log_2_^3^) HI titer

At baseline 14/40 of the chicken in the unvaccinated control, 19/37 of chicken in the drinking water group, 19/37 of chicken in the eye drop group and 20/40 chicken in the spray group had antibody titer ≥ log_2_^3^. Two weeks after the first vaccination the proportion of chicken with antibody titer ≥ log_2_^3^ rose to 23/37, 30/37 and 29/40 in the group vaccinated via drinking water, eye drop and spray, respectively (Table 4). At this time the proportion of chicken with antibody titer ≥ log_**2**_^3^ in the unvaccinated control group was 16/40. Two weeks after booster vaccination the proportion of chicken with antibody titer ≥ log_**2**_^3^ slightly increased in the group vaccinated via drinking water and spray. The proportion of chickens with antibody titer ≥ log_2_^3^ remained low in the unvaccinated control group.

**Table 4:** The proportion of chickens with HI titer ≥ log_2_^3^ among the four experimental groups

Treatment Group	Number of chickens (%) with HI log2^3^ ≥ 3.0			
	N	Day 15	N	Day 31
Control	40	17(42.5%)	40	16(40%)
Drinking water	37	23(62.16%)	37	27(72.97%)
Eye drop	37	30(81.08%)	37	30(81.08%)
Spray	40	29(72.5%)	40	31(77.5%)

### Survival of chicken after challenge infection

After infection with virulent virus, chicken in the vaccinated group showed 100% survival while 60% of the unvaccinated controls died after showing typical clinical signs of ND. Two chicken from the group vaccinated via drinking water had shown mild clinical signs of ND but both recovered well after a week. The number of chicken owned by farmers in the study area showed decreased mortality for household who vaccinate their chicken. At the beginning of this experiment 12 households owning a total of 151 chickens were registered of which 4 household were willing to participate in the study. In these households the average number of chicken per household dropped from 12.58 to 4.08 at the end of the study. In contrast 16 households owning 337 chickens (21.06 chickens per household) who got their chicken vaccinated had 296 (18.5 chickens per household) at the end of the study showing the impact of vaccination (Table 5).

**Table 5:** Number of chicken owned and survived at the end of the study period in Minjar-Shenkora district

	No. owned	No. died	No. alive	Survival
Unvaccinated				
Total	151	102	49	32.45%
Average/household	12.58	7.75	4.08	
Vaccinated group				
Total	337	41	296	87.83%
Average/household	21.06	2.56	18.5	

## Discussion

Newcastle Disease is a highly virulent disease of poultry which can devastate the entire flock in short period of time. This was evident from frequent outbreaks affecting several farms in many areas during this study. Besides, the higher proportion of chickens with mean antibody titer greater than the protective level at the baseline shows exposure of the chickens to the virus as there is no vaccination in the study area. The antibody titer declines through time in unvaccinated group suggesting the need for vaccination. That means control of ND needs to be a priority issue in the achievement of food security and reduction of poverty in Ethiopia. Vaccine delivery that is suitable for smallholder farmers is important since biosecurity issues are difficult to achieve in rural settings. In this study, oral delivery of ND I2 vaccine via drinking water and spray were compared with the recommended route of vaccination (eye drop method).

The results showed that chickens vaccinated with ND I2 via drinking water and spray elicited anti-NDV antibody level sufficiently higher than the protective level (log_2_^3^), against virulent field virus [9, 12]. That is, the level of protection as shown by the antibody titer conferred by these routes of vaccination is comparable to the antibody level elicited by the eye drop method. This shows that smallholder farmers can choose among the methods of vaccination that suits their particular farming systems and needs. Elsewhere, it was shown that chickens vaccinated via oral route elicited good protection level even though booster vaccination was needed 2–4 weeks later [6, 15].

The proportion of chickens having antibody titer ≥log_2_^3^ after the first and booster vaccinations in the group vaccinated via drinking water and spray is comparable to that of chickens vaccinated using eye drop method. Particularly the proportion of chickens with antibody titers greater than log_2_^3^ after the booster vaccinations was greater than 70%, the level of herd immunity to ensure control of outbreaks. This observation is in consent with the earlier reports of Nassir et al. [13] and Reta et al. [14] in Ethiopia. Similarly, it agrees with the reports made elsewhere in the world such as the findings of Wegdan et al. [16] who used eye drop method and drinking water to deliver ND I2 vaccines in chicken. The protection level observed in chickens vaccinated by spray is also in agreement with earlier reports of Tadios et al. [17]. The high level of protection offered by vaccination via spray could be due to higher chance of getting the vaccine virus through natural routes of infection such as the eye and nostrils. Our observation shows that oral delivery of ND I2 vaccine via drinking water, which can be carried out by farmers themselves and spray with locally available materials can provoke sufficient immunity comparable to eye drop method. Literature shows that oral administration of ND vaccines primarily provokes mucosal immunity [18]. This is important to confer protection against NDV, which is often acquired either by inhalation or ingestion or both [19].

The 100% survival observed in chickens vaccinated via drinking water, spray and eye drop methods compared to the control group after challenge infection indicates that vaccination with ND I2 vaccine can reduce mortality significantly. It reduces mortality at least by 60% in vaccinated chicken. This has important implication in terms of food security and financial revenue for smallholder chicken farmers. This is further evident from the drop in average number of chickens per household from 12.5 to 4.08 in farmers who did not vaccinate their flock. In all households who got their chicken vaccinated whether they were involved in the experiment or not the average number of chicken per households only dropped from 21.06 to 18.5. This implies that vaccination against ND with ND I2 vaccine via suitable delivery route can significantly reduce mortality and maintain flock size and ultimately contribute to household income. In consent to our observation previous authors showed that vaccination significantly increased chicken number and egg consumption per household in vaccinated village compared to control villages [4]. Women and children can particularly benefit from such vaccinations. In agreement with our findings Nasser et al. [13] and Musa et al. [20] reported 100% protection in chickens vaccinated via drinking water and eye drop following challenge with virulent virus. Reta et al. [14] also reported 100% protection in chickens vaccinated with ND I2 via drinking water after infection with the same strain of virus used in this study. Tadios et al. [17] also reported 100% survival after challenge infection with the same strain of virus in chickens vaccinated by spray. In Vietnam, after extensive laboratory and field trials in village chickens, ND I2 has been officially recognized as the ND vaccine for village chickens [8]. In Tanzania, it has been shown to offer protection for at least 2 months after vaccination [21]. Field records in Mozambique indicated that ND I2 vaccine provides approximately 80% protections in the face of outbreaks [6].

Notwithstanding the small number of households and chickens included in this study, the results showed that ND I2 vaccine provoke similar level of antibody production in indigenous and exotic chickens. In consent to our observation Nega et al. [22] reported similar level of protection in indigenous and exotic chickens after vaccination with ND I2 vaccine. This is important finding for poultry producers and veterinary personnel since information on the efficacy of ND vaccines in indigenous chicken is scarce. Regular vaccination of village chickens with ND I2 vaccine is suggested to reduce the circulation of virulent ND viruses and their spill over to commercial farms.

## Conclusions

In conclusion, ND I2 vaccine administered via drinking water and spray provoked protective antibody level and survival after infection with virulent virus similar to the eye drop method under smallholder farmers’ situation. The level protection conferred was similar in indigenous and exotic chickens reared by smallholder farmers in the area. Therefore, smallholder farmers can choose the suitable vaccine delivery routes to prevent outbreaks of ND. Vaccination can make a vital contribution to the improvement of household food and financial security in smallholder farmers.

## Abbreviations

EID: Embryo Infective Dose
HI: hemaglutination Inhibition
ND: Newcastle Disease
NVI: National Veterinary Institute
